# Response projected clustering for direct association with physiological and clinical response data

**DOI:** 10.1186/1471-2105-9-76

**Published:** 2008-01-31

**Authors:** Sung-Gon Yi, Taesung Park, Jae K Lee

**Affiliations:** 1 Department of Statistics, Seoul National University, Silim-dong, Kwanak-gu, Seoul, 151-747, Korea; 2 Division of Biostatistics and Epidemiology, University of Virginia, Charlottesville, VA 22908, USA

## Abstract

**Background:**

Microarray gene expression data are often analyzed together with corresponding physiological response and clinical metadata of biological subjects, e.g. patients' residual tumor sizes after chemotherapy or glucose levels at various stages of diabetic patients. Current clustering analysis cannot directly incorporate such quantitative metadata into the clustering heatmap of gene expression. It will be quite useful if these clinical response data can be effectively summarized in the high-dimensional clustering display so that important groups of genes can be intuitively discovered with different degrees of relevance to target disease phenotypes.

**Results:**

We introduced a novel clustering analysis approach, *response projected clustering *(RPC), which uses a high-dimensional geometrical projection of response data to the gene expression space. The projected response vector, which becomes the origin in the projected space, is then clustered together with the projected gene vectors based on their different degrees of association with the response vector. A bootstrap-counting based RPC analysis is also performed to evaluate statistical tightness of identified gene clusters. Our RPC analysis was applied to the *in vitro *growth-inhibition and microarray profiling data on the NCI-60 cancer cell lines and the microarray gene expression study of macrophage differentiation in atherogenesis. These RPC applications enabled us to identify many known and novel gene factors and their potential pathway associations which are highly relevant to the drug's chemosensitivity activities and atherogenesis.

**Conclusion:**

We have shown that RPC can effectively discover gene networks with different degrees of association with clinical metadata. Performed on each gene's response projected vector based on its degree of association with the response data, RPC effectively summarizes individual genes' association with metadata as well as their own expression patterns. Thus, RPC greatly enhances the utility of clustering analysis on investigating high-dimensional microarray gene expression data with quantitative metadata.

## Background

Microarray expression profiling has been widely applied to biological studies because of its ability to simultaneously examine tens of thousands of gene expression patterns. Microarray experiments have also proven to be quite useful for investigating associations between genes and physiological and clinical response measurements of many human diseases [1-3]. In particular, unsupervised learning techniques such as hierarchical clustering analysis have become one of the most commonly-used techniques for analyzing microarray data since these techniques can effectively summarize high-dimensional gene expression data in a two-dimensional color-coded cluster heatmap based on many genes' expression associations [4]. Several other clustering techniques such as k-means clustering, self-organization maps, and gene shaving have been used for microarray data analysis [5-7]. The main objective of these clustering analyses, however, has been to summarize the expression pattern associations among genes, but not the direct association with interesting physiological response data on study subjects.

Several supervised learning and statistical modeling approaches have also been used to analyze the gene expression data along with other response variables such as treatment group variables [8], clinical response data such as survival times [9], and Bayesian regression modeling [10]. However, these approaches are often based on the dichotomization of quantitative response data, resulting in significant loss of information. Furthermore, these methods are mainly used for the discovery of gene factors and prediction models between different response groups and cannot provide high-dimensional association information between genes and response variables.

**Figure 1:**
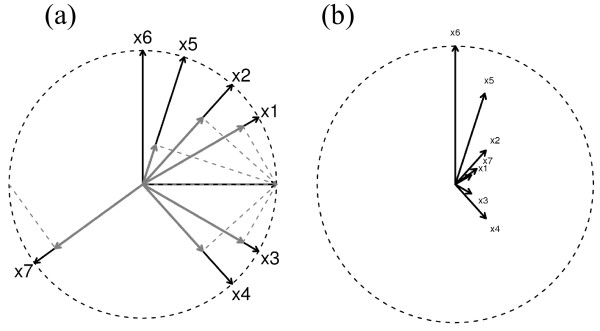
**RPC projection example for 7 gene expression variables (x_1_, ..., x_7_) and a response variable (y)**. (a) Grey arrows represent projected gene expression vectors by the response variable. The remaining fractions (dark arrows) are then used for our RPC analysis. (b) The remaining fraction (dark) arrows are redrawn to be centered at the origin. The response variable y becomes the origin in this space. Negatively-correlated genes (e.g., x_7_) can be reversely directed in this transformation by multiplying their sign (-1) of correlation.

In this study, we propose a novel clustering analysis approach, the so-called response projected clustering (RPC), which accounts for both the relationships among gene expression patterns themselves and their association with response data. This RPC approach is motivated by a relatively simple geometrical observation that a relevant response vector can be projected to each gene vector in their high-dimensional space to reflect each gene's association with the response data prior to the clustering analysis. For RPC analysis, all gene or response vectors are first standardized (so that the mean and variance are 0 and 1). The response vector is then projected into each gene so that its resulting projection resides in each gene's subspace proportional to the association strength with the response variable, not changing each gene vector's direction (so expression pattern) in the high-dimensional gene expression space (grey arrows in Fig. 1a). Clustering analysis on the remaining fractions of the genes (so that the genes with higher associations with the response variable have shorter lengths from the origin; dark arrows in Fig. 1a, which are redrawn in Fig. 1b) is then performed in the projected gene space based on their pairwise Euclidean distances. RPC thus transforms each gene expression vector into the new variable that reflects its degree of association with the response data. In this transformation, more highly-correlated genes with the response variable will have closer distances from the origin (response vector) and each other (even though they were originally relatively far apart) since they are shrunk toward the origin (Fig. 1b). Note that the response vector itself becomes the origin in this projection and that it is clustered together with other gene vectors which directly shows which groups of genes are highly associated with the response metadata. Note also that the genes initially highly correlated with each other and associated with the response variable in a similar degree will maintain their close distance and association even after this projection.

Avoiding unstable clustering patterns due to small changes of input data orders and heuristic clustering algorithms, we further refined RPC by using bootstrap-based counting measures in order to obtain robust clustering patterns with statistical significance. Our RPC method is applied to the drug activity data of *in vitro *growth inhibition by docetaxel and microarray data on the NCI-60 cancer cell lines [11] and the microarray study for atherogenic macrophage differentiation to foam cells [12].

## Results

### Docetaxel chemosensitivity and microarray gene expression data on the NCI-60 cancer cell lines

Docetaxel is one of the most widely-used anti-neoplastic chemotherapeutic compounds to treat various tumors such as breast, non-small cell lung, gastrointestinal (stomach), and prostate cancers [13]. Major target genes of docetaxel are known to be BCL2 and TUBB1. However, because this compound was originally derived from a natural extract (bark of the Pacific yew tree), its complete molecular chemosensitivity mechanisms and pathways are not completely understood [2].

In our current application, we use *in vitro *drug activity data of docetaxel on the NCI-60 cancer cell line panel, so-called GI50 (50% growth inhibition dose concentration in two-day assays) [11], together with publicly-available NCI-60 genome-wide expression profiling data of Affymetrix HG-U133A [14]. The NCI-60 cell line panel consists of nine cancer subtypes: lung, colon, breast, ovarian, leukemia, renal, melanoma, prostate, and central nervous system cancers. All microarrays are normalized by IQR-normalization which is a method that Q1 and Q3 of all microarrays have the same value [15].

**Figure 2:**
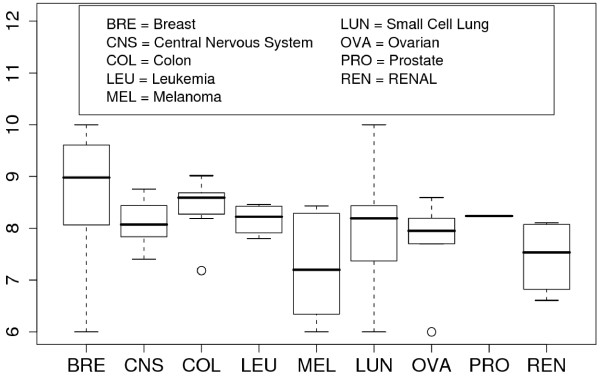
**Ranges of -log(GI50) values for nine NCI-60 cancer subtypes**. The higher the range, the more sensitive the cancer subtype to docetaxel.

Fig. 2 shows the box-plots of -log(GI50) values for the nine cancer subtypes of NCI-60. As shown, breast, non-small lung, colon, and prostate cancer cells were generally sensitive to this compound whereas melanoma and renal cancer cell lines were less sensitive. Note that the NCI-60 gene expression profiling data were obtained prior to the docetaxel treatment but we assumed that there were innate molecular expression signatures that were highly correlated with the docetaxel chemosensitivity as often found in other studies [16].

**Figure 3:**
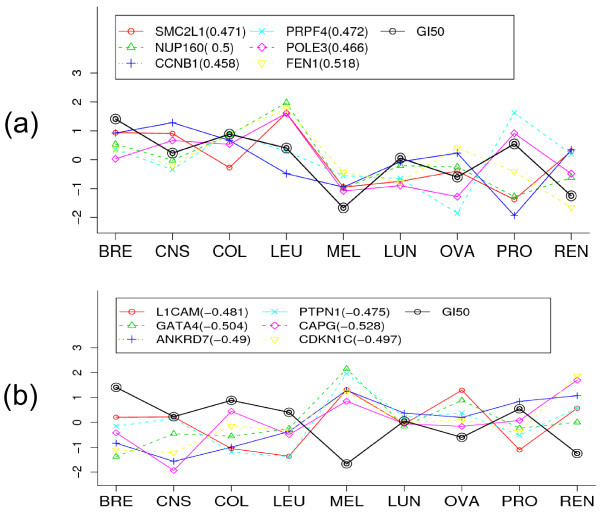
**Expression profiles of 12 correlated genes with docetaxel chemosensitivity**. (a) Six top positively-correlated genes and (b) Six top negatively-correlated genes.

We first identified genes that were strongly correlated with the GI50 values of docetaxel on NCI-60. Fig. 3 shows the top six genes' expression patterns which were either positively (with p-value < 0.0003) or negatively (with p-value < 0.0002) highly-correlated with the GI50 values. As shown in this figure, these genes' expression patterns are somewhat different – some were lowly correlated to each other, potentially implying different molecular mechanisms of the drug mechanisms of action. Thus, this simple correlation-based discovery could provide highly-correlated genes with drug response data but it was not possible to directly understand and explore these genes' interactive functional relationships with the drug's chemosensitivity. We thus applied RPC to project the NCI-60 drug activity data into its expression profiling data.

### RPC Analysis on NCI-60 Data

**Figure 4:**
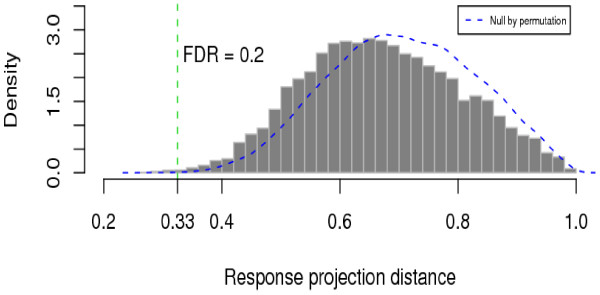
**RPC distance**. Distributions of the observed (histogram) and permutated null (dashed smooth line) RPC distances.

After we standardized gene expressions and GI50 as described in the Methods section, we derived the response projected shrinkage factors between GI50 and gene expression data with 1 - $\sqrt{\left|r\right|}$ for all the genes (histogram bars; Fig. 4) and the null distribution obtained by 1000 permutations of labels in the drug sensitivity data (dashed line; Fig. 4). *r *is the correlation between response GI50 and each gene expression. As shown, the observed distribution of the RPC projection distances is skewed toward 1 and a relatively small number of genes were identified with statistically short distances. For example, 19 genes were selected with a false discovery rate (FDR) 0.2 or less (or an RPC distance threshold 0.33 or shorter). This FDR threshold is somewhat large, but 80% of the identified genes would still be biologically relevant to the drug activity (GI50); no gene was found with FDR < 0.05. Table 1 shows the list of these 19 selected genes, their RPC distances, and functional information.

**Table 1:** The 19 selected genes by RPC. The 19 selected genes by RPC FDR < 0.2 for docetaxel chemosensitivity on the NCI-60 cell lines.

Gene symbol	Gene description	RP distance	FDR
CAPG	capping protein (actin filament), gelsolin-like	0.274	0.168
FEN1	flap structure-specific endonuclease 1	0.28	0.168
GATA4	GATA binding protein 4	0.29	0.168
NUP160	nucleoporin 160 kDa	0.293	0.168
CDKN1C	cyclin-dependent kinase inhibitor 1C (p57, Kip2)	0.295	0.168
ANKRD7	ankyrin repeat domain 7	0.3	0.176
L1CAM	L1 cell adhesion molecule	0.307	0.176
PTPN1	protein tyrosine phosphatise, non-receptor type 1	0.311	0.176
SNRPN	small nuclear ribonucleoprotein polypeptide N	0.312	0.176
PRPF4	PRP4 pre-mRNA processing factor 4 homolog (yeast)	0.313	0.176
ABCB1	ATP-binding cassette, sub-family B (MDR/TAP), member 1	0.314	0.176
SMC2L1	structural maintenance of chromosomes 2-like 1	0.314	0.176
SFT2D2	SFT2 domain containing 2	0.315	0.176
POLE3	polymerase (DNA directed), epsilon 3 (p17 subunit)	0.317	0.176
RAB5B	RAB5B, member RAS oncogene family	0.318	0.176
CCNB1	cyclin B1	0.324	0.193
DGKZ	diacylglycerol kinase, zeta 104 kDa	0.324	0.193
PPM1A	protein phosphatase 1A (formerly 2C), magnesium-dependent, alpha isoform	0.325	0.193
CNIH3	cornichon homolog 3 (Drosophila)	0.325	0.193

**Figure 5:**
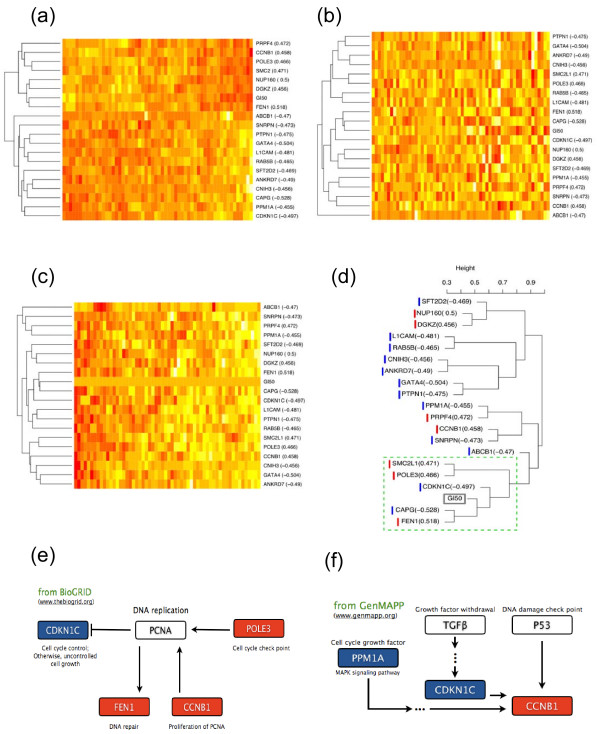
**Heatmaps and clustering dendrogram**. (a) hierarchical clustering with the correlation distance, (b) hierarchical clustering with the absolute correlation, (c) RPC analysis both for 19 genes and the docetaxel drug activity data (GI50), (d) the dendrogram of RPC analysis is shown with the branch lengths, (e) genes involved in the DAN replication pathway, (f) genes involved in the cell cycle growth factor and checkpoint pathway.

To examine the relationships among these selected genes themselves and with the drug sensitivity GI50 values, we performed the standard hierarchical clustering analysis with correlation distance (Fig. 5a), the standard hierarchical clustering with absolute value of correlation (Fig. 5b), and the RPC analysis (Fig. 5c). Note that the clustering analysis was performed among genes but not among arrays since the clustering order among the conditions was not very informative in this analysis. Fig. 5a, 5b and 5c were the heatmaps using hierarchical clustering with complete linkage. In Fig. 5a, other than a few genes (DGKZ, FEN1, NUP160) that were highly correlated with the drug activity data, most other genes were clustered based on their own gene expression associations, especially negatively and positively correlated genes separately. In Fig. 5b, FEN1 were clustered with GI50; however, DGKZ and NUP160 were clustered with other genes similar to the results of Fig. 5a. On the contrary, the RPC heatmap (Fig. 5c) shows that gene subclusters have gradually weaker associations with drug activity as they are away from the drug (response) vector. Furthermore, L1CAM, CDKN1C, and FEN1, which have been reported to be relevant to breast cancer – the most sensitive subtype to docetaxel among the nine NCI-60 cancer subtypes – were clustered just next to the drug vector. Also, CAPG and FEN1, which showed the highest correlation with GI50, were clustered together with this drug in this RPC analysis whereas CAPG was clustered in a completely different branch from the drug in the standard clustering analysis. Also note that both positively and negatively-correlated genes were well clustered together in this RPC analysis if they were highly associated to each other.

For the above selected genes, we also obtained their Gene Ontology (GO) information and further analyzed them using GOstat for evaluating statistical significance of overrepresented functional and molecular mechanisms [17]. The majority were found to belong to the molecular component of intracellular membrane-bound organelle (GO:0043231): CAPG, CCNB1, CDKN1C, FEN1, GATA4, SNRPN, DGKZ, SMC2L1, NUP160, POLE3, RAB5B, PTPN1, and PRPF4. We found that one of the known target genes of docetaxel, TUBB1, belongs to the same intracellular organelle category even though this target itself was not significant on the NCI-60 data. Many of these genes have also been found to be quite relevant to carcinogenic mechanisms. For example, L1CAM, GATA4, CCNB1, CDKN1C, and FEN1 have been reported for their association with breast cancer: L1CAM was shown to inhibit the growth of breast carcinoma cells [18]. GATA4 was reported to regulate aromatase PII promoter activity in breast cancer cells [3]. cAMP-responsive gonad-type PII promoter ultimately leads to increased intratumoral estrogen production and tumor growth. CCNB1 was reported to be upregulated in MCF-IR20 breast cancer cells by microarray experiment and to significantly reduce the clonogenic survival of MCF-IR20 cells [19]. CDKN1C showed a loss of heterozygosity for 11p15.5. 11p15.5 is an important tumor-suppressor gene region showing loss of heterozygosity in Wilms tumor, rhabdomyosarcoma, adrenocortical carcinoma, and lung, ovarian, and breast cancer [20]. FEN1 was shown to be repressed in E2 in ER-positive breast cancer cells [21]. ABCB1 is especially found to be highly relevant to the docetaxel response. It was reported that resistance arose by the overexpression of drug efflux pumps including MDR1 (P-glycoprotein/ABCB1) when docetaxel is medicated [22]. PPM1A, SNRPN, RAB5B, and CAPG were also reported to be related to cancer [23-25].

We performed the bootstrapping-based RPC analysis as described in the Methods section in order to obtain more statistically consistent subclusters in Fig. 5d. For the identified gene subclusters in this figure, we investigated several pathway databases to understand whether some of these subclusters of genes were relevant to certain carcinogenic pathway mechanisms. Interestingly, a subcluster of four genes – CDKN1C, FEN1, CCNB1, and POLE3 – were found to be directly associated with the PCNA pathway (proliferating cell nuclear anigen) which is relevant to DNA replication and cell cycle control/check point (Fig. 5e). The other subcluster of PPM1A, CDKN1C, and CCNB1 was also found to belong to the pathway of cell cycle growth factor and damage check points (Fig. 5f). Thus, it will be quite interesting to further investigate functional and pathway mechanisms of some of these tightly-clustered genes.

### RPC analysis for PPARγ during macrophage differentiation in atherogenesis

A microarray gene expression study was performed to identify novel atherogenic mechanisms involved in macrophage (MΦ) differentiation to foam cells at the University of Virginia [12]. In this experiment, human monocyte-derived macrophages (MDM) were incubated with different types of low density lipoproteins (LDL) conditions such as naïve LDL, oxidized LDL (OxLDL), and minimally modified LDL (mmLDL), which provide quite different microenvironments in atherogenesis. In this microarray experiment, peroxisome proliferator-activated receptor type γ (PPARγ), which plays important roles in atherogenesis and is a molecular target for pharmaceutical products such as Avandia^® ^for treating cardiovascular complication among type 2 diabetic patients (GlaxoSmith Kline, Inc.), was found to be highly upregulated by OxLDL and naïve LDL during the macrophage differentiation to foam cells. This selective regulation again demonstrates that PPARγ is highly relevant to atherogenesis, necessitating more targeted investigation on this gene under its respective cellular environments. However, PPARγ, as a transcription factor has been found to interact with many different genes, and its complete pathway mechanisms in atherogenesis still need to be carefully investigated associated with this gene's expression patterns on different microenvironments.

**Figure 6:**
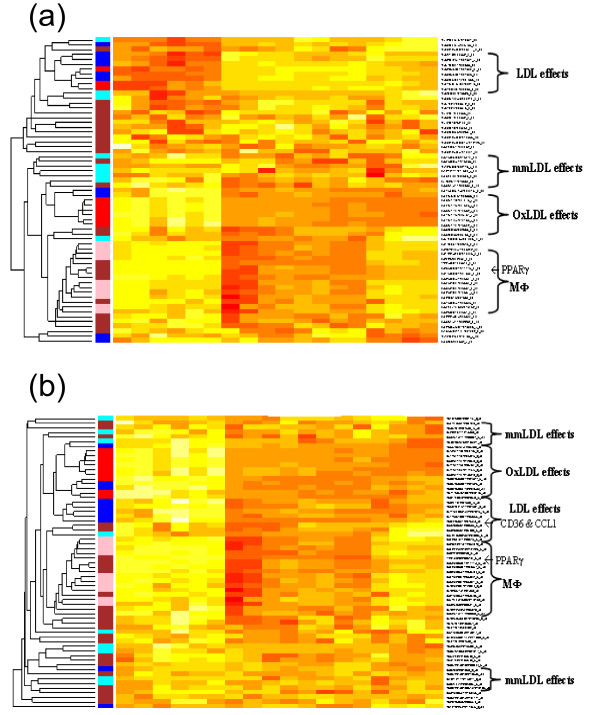
**RPC analysis for PPARγ on macroarray data during macrophage differentiation to foam cell**. (a) standard hierarchical clustering, and (b) RPC analysis. Genes are colored based on their known relevance in LDL (blue), OxLDL (red), mmLDL (turquoise), and macrophage (MΦ, pink) mechanisms.

Thus, we applied our RPC approach to the macrophage differentiation microarray data as if the gene expression values of PPARγ were response data in order to find the gene networks closely associated with this gene factor (Fig. 6). In order to remove random genes clustered with other biologically relevant genes, we preselected genes based on the significance of their differential expression among different LDL conditions with FDR < 0.05 [12]. The standard clustering analysis led to gene clusters with PPARγ based simply on each gene's correlation with other genes or PPARγ's correlation with genes (Fig. 6a). Many lowly-correlated genes with PPARγ, e.g., FEZ2 (r = 0.06), TPT1 (r = 0.19) are closely clustered with it whereas highly negatively-correlated genes, e.g. INSIG1 (r = -0.89) and CCL1 (r = -0.84) are found further away from it. On the contrary, in the RPC analysis, many genes highly correlated with PPARγ such as apoE, LPL, CD36, MT1, and IL1B are tightly clustered by themselves and closely clustered with it (Fig. 6b). PPARγ is also closely clustered with P8, PPARβ, and ABCG1 which are well-known for their roles in atherosclerosis. Lowly-correlated genes are assigned away from PPARγ gradually in this RPC analysis, and both positively and negatively highly-correlated genes are closely clustered with this gene despite their opposite expression directions.

PPARγ has also been reported to regulate many fatty acid factors during the form-cell formation including a group of fatty acid regulation genes such as CD36, ABCA1, apoE, and LPL [26]. In our RPC analysis, we could effectively identify their tight associations with PPARγ and discover novel gene factors such as CCL1 and IL1B which are also known to play a role in atherogenesis but have not been reported for any direct association with PPARγ. For example, the two transcripts of CD36 (correlation with PPARγ r = 0.65, 0.58) are tightly clustered with CCL1 (r = -0.84) which implies their close functional association in the opposite direction, or potentially inhibition. Note that these clustering results could not be observed by a standard clustering analysis. Overall, applying RPC to PPARγ expression patterns on the MΦ-differentiation microarray data, genes relevant to atherogenic PPARγ functions, e.g., LDL (blue) and OxLDL (red) groups move closer to the PPARγ gene whereas the groups of genes less relevant to PPARγ, e.g., mmLDL (turquoise) groups move away from it.

## Discussion

We introduced a novel clustering analysis approach here – response projected clustering (RPC) that can simultaneously summarize associations both with important physiological response data and with gene expression patterns themselves. The RPC method effectively performs such an integrated analysis by directly projecting response data into the high-dimensional gene expression vectors. We believe that since clustering analysis plays a significant role in exploring coexpression patterns of a large number of genes in microarray profiling data, the RPC approach will be quite useful by examining such high-dimensional data simultaneously with those genes' association with the response data. Using a bootstrapping-based clustering measure, we also performed RPC analysis based on statistical significance of tightness of subclusters.

RPC can be utilized in many different clustering analyses to investigate high-throughput biological profiling data together with relevant physiological response data if molecular signatures exist in the profiling data highly associated with the physiological response. It can also be used for a particular gene in microarray data to investigate the gene's associated groups of other genes. We, however, suspect that the degrees of molecular association with some response data such as patient long-term survival and outcome data may be weak and noisy, and careful understanding on such an association may improve the utility of the RPC technique.

In our current study, we first applied RPC to the docetaxel drug activity and the microarray expression profiling data on the NCI-60 cancer cell lines. In this application, the selected genes (many of which were known for their carcinogenic mechanisms) were found to be quite tightly associated with DNA replication and cell cycle pathways. The above findings may suggest that this compound interferes with the DNA replication process in order to inhibit tumor cell growth; it will be interesting if the roles and functions of these genes are further investigated for their involvement in this drug activity to administer this chemotherapeutic compound more effectively in treating patients.

We showed a different utilization of the RPC approach in our second application by using the expression values of a targeted gene factor, PPARγ, as response data in order to find other genes' expression patterns and networks closely associated with this gene. In this analysis, we were able to confirm many known genes as well as novel gene factors relevant to this target gene's functions and pathways in atherogenesis. In order to apply RPC to a subset of biologically relevant genes, we preselected genes that were differentially regulated between the experimental conditions of atherogenesis with FDR < 0.05; bigger FDR cutoff values resulted in much larger numbers of genes of which clustering results were less clear and difficult to interpret. This application demonstrates well that one can utilize RPC analysis in searching gene association networks on various contexts of genome-wide expression studies associated with a particular gene factor.

In this study, we used 1 - $\sqrt{\left|r\right|}$ as an RPC (projection) distance based on correlation (association) between the gene expression and drug activity data (e.g., Pearson, Spearman, or binary correlation). Of course, any projection distance of *c*-*f*(*r*) formed with a monotone function *f *can be used as such a distance if such a projection transformation can effectively discriminate different degrees of association with response data among candidate molecular signatures. Also note that the RPC transformed distance, directly derived from the RPC geometrical projection, can be modified into an even simpler form such as:

*d*_*RPC*_(**x**_*g1*_, **x**_*g2*_) = [1 - *f*(|*r*_*1*_|) *f*(|*r*_*2*_|)] || **x**_g1 _- **x**_g2_||,

when **x**_*g1 *_= {*xg1*1,...,*xg1n*} and **x**_*g2 *_= {*xg2*1,...,*xg2n*} are the *g*_*1 *_and *g*_*2 *_gene vectors, respectively. The *r*_*1 *_is the correlation between the *g*_*1 *_gene vector and response vector and the *r*_*2 *_is between the *g*_*2 *_gene vector and response vector. We also note that several different clustering algorithms have been explored in our preliminary studies such as single, complete, average linkages (data not shown). While they show slightly different tree structures, the tightly clustered genes were found to be consistent. Thus, the clustering results presented here use the average linkage algorithm.

Other forms of modification are certainly possible which may deserve a full comparison study both by simulation and practical application in a future study. More generally, RPC can be applied with different measures of association beyond correlation evaluation if the association between the biological profiling data and response data can be identified with a different measure, e.g. SNP data with linkage association scores. These different functions and algorithms need to be further investigated in the future. Also note that we introduced our RPC algorithm using hierarchical clustering but our RPC projection can be applied to other clustering algorithms such as k-means, SOM, and others. Finally, we note that RPC application will be more difficult if the degrees of molecular association are weak and noisy with some response data such as patient long-term survival and outcome data. In these cases, careful understanding on such association may improve the utility of the RPC technique.

## Conclusion

We introduced a novel clustering analysis approach here – response projected clustering (RPC) – that can simultaneously summarize associations both with important physiological and clinical response data and with gene expression patterns themselves. RPC can be considered as an enhanced integration of the unsupervised learning with supervised learning techniques, effectively performing such an integrated analysis by directly projecting response data into the high-dimensional gene expression vectors. Using its simple projection transformation, the RPC approach allows one to effectively examine high-dimensional gene expression data simultaneously with relevant response data or with a specific gene target which would be extremely useful in many biomedical gene expression studies.

## Methods

### RPC shrinkage distance and analysis

We assume all microarray data are IQR normalized (among different chips) prior to our analysis. Suppose there are *n *subjects and *p *genes on microarray profiling together with *n *subjects' response data **y **= {*y*1,...,*yn*}. Let **x***i *= {*xi*1,...,*xin*} be an *n*-dimensional vector of the *i*th gene's expression, *i *= 1,...,*p*. We first standardize each of these response and expression vectors (so that the mean and variance are 0 and 1) to have the same scale (on a unit sphere). Denote the new standardized variables as:

$$\begin{array}{cc} y_{j}=\frac{y_{j}-\bar{y}}{\sqrt{\sum {\left(y_{j}-\bar{y}\right)}^{2}}}, & x_{ij}=\frac{x_{ij}-{\bar{x}}_{i}}{\sqrt{\sum {\left(x_{ij}-{\bar{x}}_{i}\right)}^{2}}}, \end{array}$$

*i *= 1,...,*p*, *j *= 1,...,*n*. Note that the same notations are used for these standardized vectors as the original vectors because there is no loss of information after this standardization if pairwise distances are evaluated based on their co-expression (or association) patterns by, e.g., Pearson correlation for clustering analysis.

For the projection of response data into gene variables, we then calculate the inner product between the standardized response vector and each standardized gene expression vector:

ri=〈y,x〉i=∑j=1nyjxij,i=1,...,p

The resulting inner product is the cosine value of the internal angle (in the *n*-dimensional space) between the response vector and each gene vector. Note that this value is thus the projected magnitude of the response vector to each gene vector; it is also the correlation between the two vectors. For example, if the two vectors have an inner angle close to 0° (or 180°) or a strong correlation, this value will be close to 1 or -1. Without changing its direction, each gene vector is then resized with RPC shrinkage factor *s*_i_:

**x**_i_* = *s*_*i *_**x**_*i*_, where *s*_*i *_= (1-*r*_*i*_), *i *= 1,...,*p*

(The dark fractions of arrows in Fig. 1a). Note again that the response vector itself then becomes the origin (because *r *= 1) and a gene vector with a higher correlation (≈1) with the response vector will have a bigger shrinkage effect (so closer to the origin).

If one wants to group both negatively and positively-correlated genes together as long as they are highly correlated to each other, the shrinkage factor can be obtained with a general monotone function *f *on the absolute magnitude of *r*_*i *_as:

**x**_i_* = *s*_*i *_**x**_*i*_, where *s*_*i *_= 1 - *f*(|*r*_*i*_|), *i *= 1,...,*p*

For example, **x**_i_* = (1 - $\sqrt{\left|r_{i}\right|}$) **x**_i _with *f*(x) = (1 - $\sqrt{\left|x\right|}$), = (1-$r_{i}^{2}$) **x**_*i *_with *f(x) *= x^2^, or = {c-log|(1+*r*_*i*_)/(1-*r*_*i*_)|} **x**_i _with Fisher's z-transformation where c = max log|(1+*r*)/(1-*r*)| if *r *< 1. These transformations may be used in order to make the RPC analysis more sensitive to small differences in a highly-skewed correlation distribution. In our current study, we use **x**_i_* = (1 - $\sqrt{\left|r_{i}\right|}$) **x**_*i*_, *i *= 1,...,*p*, because this choice was found to be sensitive in the range of a commonly-observed value of |*r*_*i*_| around 0.5.

The clustering distance between two gene expression vectors, say **x**_*g1 *_and **x**_*g2*_, is thus calculated based on their Euclidean distance (Fig. 1b):

*d*_*RPC*_(**x**_*g1*_, **x**_*g2*_) = || (1 - *f*(|*r*_*1*_|) **x**_g1 _- (1 - *f*(|*r*_*2*_|) **x**_g2 _||

where *r*_*1 *_and *r*_*2 *_are the inner products (or correlations) of *x*_*g1 *_and *x*_*g2 *_with the response vector **y**. Note that the original Euclidian distance was *d*(*x*_*g1*_, *x*_*g2*_) = ||*x*_g1 _- *x*_g2_|| prior to the RPC transformation. More discussions on this distance will be found later. Therefore, in this RPC analysis, the gene vectors highly associated with the response vector will have very short distances from the origin and consequently short clustering distances would be obtained between them. All other genes will be gradually clustered away from the response vector as their degrees of association with the latter weakens.

**Figure 7:**
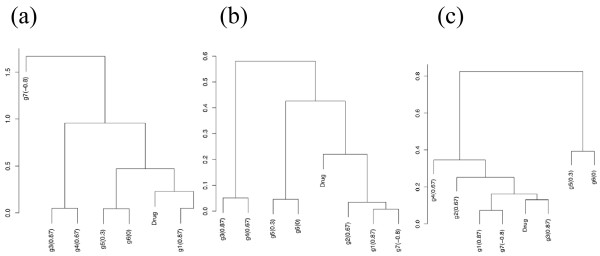
**Effects of response projected clustering**. (a) standard hierarchical clustering using the correlation, (b) hierarchical clustering using the absolute value of the correlation, (c) response projected clustering.

The utility of this RPC approach can be illustrated in a simple example as below. For example, drug response data and seven other gene expression vectors are synthetically generated with correlation coefficients 0.87, 0.67, 0.87, 0.67, 0.31, 0, and -0.81 between drug response "Drug" vector and each of the seven gene vectors g1-g7 (also refer to Fig. 1 depicted as ***y ***and ***x***_1_,...,***x***_7 _within a two-dimensional unit circle). So, g1, g3, and g7 are the most highly-correlated with the drug response, especially g7 negatively. Each of the seven genes is then shrunk based on the projected length of the drug response as in Fig. 1b. Note that if a gene like g7 is reversely correlated, the direction is also reversed in this projection. The effects of RPC are then demonstrated in Fig. 7. First, in an application of the standard hierarchical clustering algorithm to these synthetic genes, the first three pairs of the seven genes, g1-g2, g3-g4, and g5-g6 were tightly clustered together (Fig. 7a). However, this clustering does not reflect the association with the drug response; the least correlated gene g6 (correlation 0) appears just next to the drug response due to the ordering in this clustering algorithm. Furthermore, the most highly-correlated gene g3 is assigned away from it and the negatively highly-correlated gene g7 appears to be quite irrelevant to the drug response. The clustering based on the absolute correlation distance cannot yet identify all the genes highly associated with the response vector (Fig. 7b). By contrast, in the RPC analysis, both positively and negatively-correlated genes are tightly clustered with the drug response (Fig. 7c). Specifically, the correlation structure of g1 and g7, which are perfectly negatively correlated and the most highly-correlated genes with the drug response, is well reflected in this clustering. Note that this simulation example is shown to explain the mechanistic procedure and effects of RPC which may not be obvious in RPC applications in large real microarray data sets. Also note that RPC is always performed with the transformed response vector so that one knows exactly where the RPC vector falls among the gene clusters.

### Significance and consistency of RPC sub-clusters

In order to evaluate the statistical significance of each RPC gene's association with the response data, we can generate random data directly using the original microarray data by repeatedly permutating sample identities in the response data. From such a permutation-based null distribution, we can evaluate the statistical significance of each observed *d*_*i *_for the *i*th gene, *i *= 1,...,*p*, compared to *d*_*im*_, *m *= 1,...,*M *from *M *permutated samples:

$$p_{i}=\frac{\sum_{m=1}^{M}I(d_{i}\leq d_{im})}{M},\;i=1,\ldots ,p$$

The false discovery rates (FDR) from these (empirical) p-values are then derived for the multiple test adjustment [27]. We use these FDR values for selecting genes for our final clustering analysis, e.g. FDR < 0.2.

**Figure 8:**
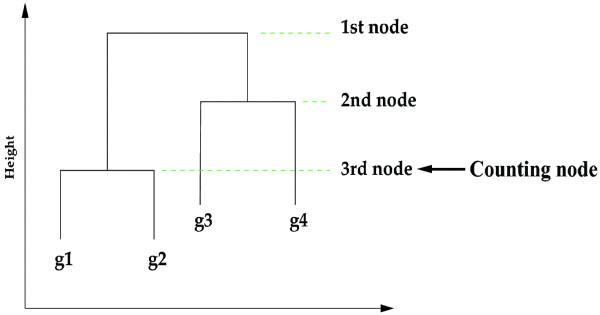
**Co-clustering counting**. The *q*-percentile node for co-clustering counting.

Due to the nature of its heuristic allocation algorithms, clustering analysis can often provide different groups of clustered genes with slightly different input data or even with different orders of genes. Statistical confidence evaluation on clustered gene groups has thus been suggested using resampling techniques such as bootstrap [28,29]. We also use a bootstrapping technique to assess the stability of our RPC clustering results among RPC selected, say, *s *genes. We obtain *B *bootstrapped samples of size *n *{***z***_***1***_^***b***^**, ..., *z***_***n***_^***b***^}, *b *= 1,...,*B *from the original *n *subjects (column vectors) {***z***_1_*, ..., *z*_n_*} with replacement where ***z***_j_* = {*x*_1i_*, ..., *x*_pi_*} is the *s*-dimensional vector of the *j*-th subject. The consistency of sub-clusters of the *s *genes can be examined from these bootstrapped samples. For example, the probability that two genes belong to a common subcluster can be assessed by counting the frequencies of their co-clustering occurrences at a particular node, e.g. 75-percentile node of each cluster dendrogram (Fig. 8).

At the *q-percentile *node of cluster dendrogram, an *s *by *s *counting table ***C***^*q *^can be constructed, each cell with the fraction of bootstrapped dendrograms in which each pair of genes were clustered together (Table 2). The higher the fraction, the more likely its corresponding pair of genes would cluster together. Therefore, using 1-***C***^*q *^as a pseudo pairwise distance matrix, we can perform the cluster analysis for *s *genes. In general, we found that a 50-percentile node well reflects the consistent co-clustering patterns of genes. This bootstrap-counting clustering algorithm can thus be summarized in the following three steps:

**Table 2:** Bootstrapped counting table. Bootstrapped counting table for co-clustering of genes

	G1	G2	G3	G4
G1	1	0.85	0.3	0.2
G2	0.85	1	0.2	0.25
G3	0.3	0.2	1	0.78
G4	0.2	0.25	0.78	1

(1) Generate bootstrapped samples of size *n *{***z***_***1***_^***b***^**, ..., *z***_***n***_^***b***^}, *b *= 1,...*B*.

(2) Apply hierarchical clustering to each of the bootstrap samples. At the *q*-percentile node, construct a counting table ***C***^*q *^across *B *bootstrapped dendrograms.

(3) Perform hierarchical clustering using a pseudo distance matrix 1-***C***^*q*^.

Thus, this bootstrap-based clustering can effectively summarize the statistical confidence on the tightness of gene clusters. Note that the height of a clustering dendrogram node then represents how strongly the members of the cluster are clustered; the closer to the bottom of the dendrogram tree, the tighter the elements in a cluster.

## Authors' contributions

SGY performed the statistical analysis and drafted the manuscript; TP participated in data interpretation and helped to draft the manuscript; and JKL conceived the statistical method for analysis and finalized the manuscript. All authors read and approved the final manuscript.
